# Oxidative Stress Mediates the Antiproliferative Effects of Nelfinavir in Breast Cancer Cells

**DOI:** 10.1371/journal.pone.0155970

**Published:** 2016-06-09

**Authors:** Maria Soprano, Daniela Sorriento, Maria Rosaria Rusciano, Angela Serena Maione, Gennaro Limite, Pietro Forestieri, Dario D’Angelo, Matteo D’Alessio, Pietro Campiglia, Pietro Formisano, Guido Iaccarino, Roberto Bianco, Maddalena Illario

**Affiliations:** 1 Department of Translational Medical Science, University of Naples Federico II, Naples, Italy; 2 Institute of Biostructure and Bioimaging (IBB) of the Italian National Research Council (CNR), Naples, Italy; 3 Department of Clinical Medicine and Surgery, Breast Unit, University of Naples Federico II, Naples, Italy; 4 Department of Emergency and Reception, Plastic Surgery Operative Unit, Hospital Center A. Cardarelli, Naples, Italy; 5 Department of Pharmacy, University of Salerno, Salerno, Italy; 6 Department of Medicine and Surgery, University of Salerno, Salerno, Italy; 7 Department of Clinical Medicine and Surgery, Oncology Division, University of Naples Federico II, Naples, Italy; National Cheng Kung University, TAIWAN

## Abstract

The discovery of the anti-proliferative activity of nelfinavir in HIV-free models has encouraged its investigation as anticancer drug. Although the molecular mechanism by which nelfinavir exerts antitumor activity is still unknown, its effects have been related to Akt inhibition. Here we tested the effects of nelfinavir on cell proliferation, viability and death in two human breast cancer cell lines and in human normal primary breast cells. To identify the mechanism of action of nelfinavir in breast cancer, we evaluated the involvement of the Akt pathway as well as the effects of nelfinavir on reactive oxygen species (ROS) production and ROS-related enzymes activities. Nelfinavir reduced breast cancer cell viability by inducing apoptosis and necrosis, without affecting primary normal breast cells. The antitumor activity of nelfinavir was related to alterations of the cell redox state, coupled with an increase of intracellular ROS production limited to cancer cells. Nelfinavir treated tumor cells also displayed a downregulation of the Akt pathway due to disruption of the Akt-HSP90 complex, and subsequent degradation of Akt. These effects resulted to be ROS dependent, suggesting that ROS production is the primary step of nelfinavir anticancer activity. The analysis of ROS-producers and ROS-detoxifying enzymes revealed that nelfinavir-mediated ROS production was strictly linked to flavoenzymes activation. We demonstrated that ROS enhancement represents the main molecular mechanism required to induce cell death by nelfinavir in breast cancer cells, thus supporting the development of new and more potent oxidizing molecules for breast cancer therapy.

## Introduction

Breast cancer is the most common type of cancer worldwide in women [1]. Despite recent advances in drug therapy, a significant proportion of breast cancer patients fail to heal for the lack of chemotherapic drugs selectivity and for the emergence of endocrine-resistance, primarily due to the activation of alternative proliferation pathways [2, 3]. In this context, the development of new drugs becomes necessary for a more effective breast cancer therapy [3–5].

Nelfinavir, initially designed to block HIV-protease [6], possesses a relevant anticancer activity by affecting many intracellular pathways involved in tumor cell proliferation and cell-death resistance. Although nelfinavir primary target is unknown, its antitumor effects have been related to several mechanisms of action: induction of endoplasmatic reticulum stress, inhibition of proteasome function, inhibition of Akt phosphorylation, and induction of autophagy [7–13]. Since Akt signaling affects different steps of cancer development [14–18], it is considered the most important nelfinavir therapeutic target. Indeed, nelfinavir-mediated inhibition of AKT phosphorylation has been associated with reduced tumor cell proliferation and increased sensitivity to ionizing radiation and chemotherapy. Therefore, nelfinavir has been tested in combination with chemo-radiotherapy for locally advanced rectal cancer [19], glioblastoma [20], head and neck carcinoma and non-small-cell lung carcinoma [21, 22]. However, the kinetic of Akt inhibition is cell line specific [11, 23–25], hence we evaluated Akt involvement in nelfinavir anticancer activity in breast cancer.

It is established that the nelfinavir maximum plasma concentration of 3-4mg/l in HIV-positive patients [26] is also able to inhibit tumor cell growth. However, it has been reported that in HIV-positive patients, long-term treatment with nelfinavir can trigger side effects that resemble the metabolic syndrome [27]. It has been proposed that drug-induced oxidative stress plays a central role in this process. The link between HIV-protease inhibitors exposure and increased ROS production is well established both in HIV positive patients [28, 29] and in several cellular models [30–33]. ROS are produced spontaneously in the mitochondria during the oxidative phosphorylation process, or through the activation of lipoxygenase, cyclooxygenase, specific oxidoreductases, and flavoenzymes [34, 35]. Regulated ROS production is essential for several biological functions such as cell growth [36], differentiation [37], and apoptosis [38] by inducing oxidative modification of proteins involved in different intracellular pathways, thus modulating their activity or half life [39]. Conversely, high intracellular levels of ROS can determine oxidative damage to DNA, lipids, and proteins [40, 41], playing a role in the progression of several processes such as carcinogenesis or cell-death [42]. Cells often tolerate mild oxidative stress by upregulating synthesis or activity of antioxidant agents to restore the balance [39, 43] but, when ROS overcome cell antioxidant defense system, oxidative stress and subsequent macromolecular damage occur [44].

It has been well established that in cancer cells ROS production is higher than normal cells [45–47], and several studies reported the presence of markers of constitutive oxidative stress in samples from in vivo breast carcinoma [48–50]. High basal level of ROS in cancer cells makes them more vulnerable to the further increase of ROS that cause cytotoxicity, suggesting the induction of oxidative stress as therapeutic strategy. To date, the role of ROS as key-players in the molecular mechanism by which nelfinavir exerts its anticancer effect has been recently investigated [51]. Our results show for the first time that nelfinavir anticancer activity may take into account simultaneous inhibition of Akt pathway and induction of ROS production, taking advantage of their involvement in the control of cell proliferation at multiple signaling levels.

## Materials and Methods

### Cell Culture

Human breast cancer cell lines (MCF-7; MDA-MB231) were grown in Dulbecco’s modified Eagle’s medium (DMEM) containing 10 mM glucose supplemented with 10% fetal calf serum and 100 units/mL each of penicillin and streptomycin and 2 mmol/L glutamine (all from Gibco, Grand Island, NY, USA) and incubated in standard culture conditions (95% air and 5% CO_2_ at 37°C).

### Culture of human primary mammary epithelial cells

Human mammary epithelial cells (HMEC) were derived from surgical specimens from women who had undergone reductive mammoplasty, following written informed consent. For investigations involving human subjects, authors affirm that they have been carried out in accordance with the Declaration of Helsinki and approved by the “Federico II University Ethical Committee” (e-mail: comitato.etico@unina.it, protocol number 159/11). Epithelial cells were culled and grown as described by Labarge and colleagues [52]. Briefly, each tissue specimen was washed extensively in phosphate-buffered saline (PBS) supplemented with 200 U penicillin, 200 μg/ ml streptomycin (Gibco) and 5 μg/ml fungizone (Sigma, Saint Louis, MO, USA), then finely minced and disaggregated for 18–20 min in 0.1% collagenase type I (Invitrogen, Carlsbad, CA, USA). Following tissue digestion, the fat supernatant tissue was removed and the tube was shaken vigorously to disaggregate any remaining large clumps. Three cell populations (epithelial breast cells, stromal breast cells and organoid substance) were then isolated using differential centrifugation. For the first 24 h, cells from the organoid and epithelial fractions were plated in 75% organoid medium (OM) to promote cell attachment. OM consisted of DMEM/F12 supplemented with 100 U/ml penicillin, 100 μg/ml streptomycin, 2 mM glutamine, 10 mM Hepes, 0.075% bovine serum albumin, 10 ng/ml cholera toxin, 0.5 μg/ml hydrocortisone, 5 μg/ml insulin and 5 ng/ml epidermal growth factor (all from Sigma, Saint Louis, MO, USA). After 24 hours medium was removed and replaced with OM. Cells were maintained in this way for the duration of the culture. To remove the fibroblasts from HMEC, differential trypsinization was performed, based on the rapid detachment of fibroblasts from the surface plastic [53].

### Reagents and inhibitors

Nelfinavir (chemical name: 3-Isoquinolinecarboxamide, N-(1,1-dimethylethyl)decahydro-2-[(2R,3R)-2-hydroxy-3-[(3-hydroxy-2-methylbenzoyl)amino]-4-(phenylthio)butyl]-(3S,4aS,8aS), was dissolved in DMSO to a final concentration of 50 mM and stored at -20°C. Its structure is well defined [54]. It was obtained through the NIH AIDS Research and Reference Reagent Program, Division of AIDS, NIAID, NIH. It was used at the indicated concentration, and added to the culture medium every 48 hours. Cycloheximide, MG-132, tocopherol, diphenyleneiodonium (DPI), (4-2-amino-ethyl)-benzolsulphonyl-fluoride hydrochloride (AEBSF), NG-nitro-l-arginine methyl ester (L-NAME) and allopurinol were all purchased by Sigma (Saint Louis, MO, USA) and used at indicated concentrations.

### Cell Viability Assay

Cells were seeded into 96-well plates to a density of 5x 10^3^ cells/well. After 24 hours of growth to allow attachment to the wells, nelfinavir or indicated reagents were added at different concentrations for the different time points. At the end of incubation times, PrestoBlue™ Reagent (Invitrogen, Carlsbad, CA, USA) was added directly in the culture medium for 2 hours, at 37°C in the dark. According to manufacturer’s indication the absorbance was measured at 570 nm, and the values normalized to the 600 nm values for the experimental wells. Results were expressed as percentage relative to vehicle-treated control (0.5% DMSO was added to untreated cells).

### Cell proliferation

Cells were seeded in 12-well culture plates at density of 7x10^4^ cells/well and treated with indicated reagents. The number of viable, trypan blue (Sigma, Saint Louis, MO, USA) -excluding cells was determined using a Bürker chamber every 24 hours until 6th day.

### Cell cycle analysis

For FACS (fluorescence-activated cell sorting) analysis cells were seeded in 6-well plates at density of 3x10^5^ cells/dish, and treated as indicated. MDA-MB231 and MCF-7 cells were collected and fixed over-night in ice-cold 70% ethanol at -20°C. Washed pellets were resuspended in PBS containing 10μg/ml Propidium Iodide (PI) (Sigma, Saint Louis, MO, USA), incubated for 30’ at room temperature, and analyzed for emission in PE-Texas Red channel. Samples were acquired with a CYAN flow cytometer (DAKO Corporation, San Jose, CA, USA). To remove artifacts such as doublets and aggregates from the analysis, an electronic doublet discrimination was performed using the area and width of the fluorescence PE-Texas Red pulse. Cell cycle distribution, expressed as percentage of cells in the G0/G1, S, and G2/M phases, was calculated using the SUMMIT software.

### Annexin V/PI staining

MDA-MB231 and MCF-7 cells were plated at 3x10^5^ in 6-well plates, washed with PBS and then with Annexin V Binding Buffer. After centrifugation at 2000 rpm for 3 min, cells were resuspended in 100μl of Annexin V Binding Buffer (Biolegend, San Diego, CA, USA) and incubated with 5 μl of fluorescein isothiocyanate (FITC)-conjugated Annexin V (Biolegend, San Diego, CA, USA) for 15 min at 25°C in the dark. Finally, 400 μl of Annexin V Binding Buffer and 2 μl of 500 μg/ml PI (Sigma) were added to each sample just before analysis. Samples were acquired with a CYAN flow cytometer (DAKO Corporation, San Jose, CA, USA) and analyzed using the SUMMIT software.

### Measurement of ROS intracellular levels

Breast cancer and primary normal cells were seeded in 6-well plates at a density of 3x10^5^ cells/dish, and treated with 10 μM nelfinavir at different time points. Cells were rinsed with PBS and incubated with 5μM H2DCF-DA (Calbiochem, San Diego, CA, USA) in serum-free fresh medium for 30 min at 37°C in the dark. Cells were washed, harvested and green fluorescence intensity examined by CYAN flow cytometer (DAKO Corporation, San Jose, CA, USA) analysis using the SUMMIT software.

### Lipid peroxidation analysis

Lipid peroxidation was analyzed using the parameters indicated in the Lipid Peroxidation malondialdehyde (MDA) assay kit (Abcam Cambridge, MA, USA). Briefly, cells were seeded at a density of 1x10^6^cells, treated with nelfinavir at the indicated time, lysed on ice in MDA lysis buffer and centrifuged (13000xg, 10 min) to remove insoluble material. The supernatants were placed into new vials with thiobarbituric acid solution for 60 min at 95°C and cooled in an ice bath for 10 min. The MDA- thiobarbituric acid adducts were quantified colorimetrically at 532 nm using a microplate reader.

### Superoxide dismutase activity assay

Superoxide dismutase (SOD) Activity Assay kit (Abcam Cambridge, MA, USA) was used to determine the SOD activity in breast cancer cell lines treated for 30 min, 3h, 24h and 48h with 10 μM nelfinavir. Briefly, cells were homogenized in ice cold 0.1M Tris/HCl, ph 7.4 containing 0.5%Triton X-100, 5mM β-mercaptoetanolo, 0.1mg/ml phenylmethanesulfonylfluoride (PMSF). After centrifugation (14000xg, 5 min at 4°C), supernatants were incubated with Working Solution and Enzyme Working Solution for 20 min at 37 C. SOD activity (%) was calculated as indicated in the assay kit instructions, using absorbance values at 450 nm.

### Quantitative reverse transcription polymerase chain reaction

Total RNA were extracted from MDA-MB231 cells using Trizol reagent (Invitrogen, Carlsbad, CA, USA), purificated with Qiagen RNeasy mini-kit and reverse transcribed using a High Capacity Reverse Transcriptase Kit (Applied Biosystems, Invitrogen, Carlsbad, CA, USA). Quantitative reverse transcription polymerase chain reaction (qRT-PCR) was performed using a BioRad IC5 thermo cycler (Bio-Rad laboratories, Hercules, CA, USA) using specific primers [55]:

  h-Akt1  5′-ATGAGCGACGTGGCTATTGTGAAG-3′ forward

                5′-GAGGCCGTCAGCCACAGTCTGGATG-3′reverse,

  h-Akt2  5′-ATGAATGAGGTGTCTGTCATCAAAGAAGGC-3′ forward

                5′-TGCTTGAGGCTGTTGGCGACC-3′reverse,

  h-Akt3  5′-ATGAGCGATGTTACCATTGT-3′ forward

                5′-CAGTCTGTCTGCTACAGCCTGGATA-3′reverse.

Cycle threshold values from 3 independent experiments were normalized to the internal beta-actin control. The ratio of fold change was calculated using the Pfaffl method [56].

### Western Blot and immunoprecipitation analysis

Cells were washed in PBS buffer and lysed on ice for 30 min in RIPA buffer[57, 58]. Lysates were quantified by Biorad DC protein assay. An equal amount of proteins from each sample was loaded with laemmli buffer. Protein were resolved by SDS-PAGE and transferred to an Immobilion P membrane (Millipore Corporation, Bedford, MA, USA). Membranes were blocked by incubation with PBS 0,2% tween, 5% nonfat dry milk for one hour at room temperature. The membranes were then incubated overnight with primary antibodies at 4°C, washed for 40 min with PBS 0,2% tween and incubated for one hour with a horseradish peroxidase-conjugated secondary antibodies. Finally, protein bands were detected by an enhanced chemiluminescence system (Amersham Bioscience, UK). Computer-acquired images were quantified using the ImageQuant software (Amersham Bioscience, UK).

For immunoprecipitation assay, cells were lysed in RIPA buffer and 500μg of total lysate were incubated with primary antibodies against protein of interest for one hour and with Protein G plus/protein A agarose beads (Calbiochem, San Diego, CA, USA) for other two hours. Non immune rabbit or mouse immunoglobulins G was used as control. Mouse antibodies to HSP90α/β (F-8, sc-13119, 1:1000), cyclin B (GNS1, sc-245, 1:1000), p21 (F-5, sc-6246, 1:1000), cytochrome c (2G8, sc-65396, 1:500), B-cell lymphoma 2 (Bcl-2) (C-2, sc-7382, 1:500), beta-actin (C4, sc-47778, 1:1000), rabbit to mouse double minute 2 homolog (MDM2) (N-20, sc-813, 1:1000), cyclin A (H-432, sc-751, 1:500), cyclin D (M-20, sc-718, 1:1000), cyclin E (M-20,sc-481, 1:1000), SOD1 (FL-154, sc-11407, 1:500), SOD2, Bak (G-32, sc-832, 1:500), caspase 9 (H-83, sc-7885, 1:500), estrogen receptor α (ERα) (D-12, sc-8005, 1:1000), endothelial nitric oxide synthase (eNOS) (C-20, sc-654, 1:500) and phospho-eNOS (Thr495) (sc-19827, 1:1000), goat anti-Akt (C-20, sc-1618, 1:1000) and phospho-eNOS (Ser1177) (sc-12972, 1:1000) were all purchased from Santa Cruz Biotechnology (Santa Cruz, Dallas, TX, USA). Rabbit polyclonal to phospho-Akt (Ser 473) (#4060, 1:1000), pospho- proline-rich Akt substrate 40 kDa (pPRAS40) (Thr 246) (#2640, 1:1000), phospho-Rb (Ser 807/811) (#9308, 1:1000) and catalase (#12980, 1:1000) were purchased from Cell Signaling (Danvers, MA, USA). In some experiments, the release of cytochrome c was evaluated in cytosolic extracts after mitochondria removal by western blot, as previously described[59, 60].

### Statistical analysis

All results are presented as mean ± SEM. Statistical analysis was performed with GraphPad Prism software (San Diego, CA, USA) using the unpaired Student’s *t* test, 2-tailed (for 2 groups), and 1-way ANOVA with Tukey-Kramer post hoc correction (for groups of 3 or more). *P* < 0.05 was considered to be statistically significant.

## Results

### Effect of nelfinavir on breast cells viability and proliferation

**Fig 1:**
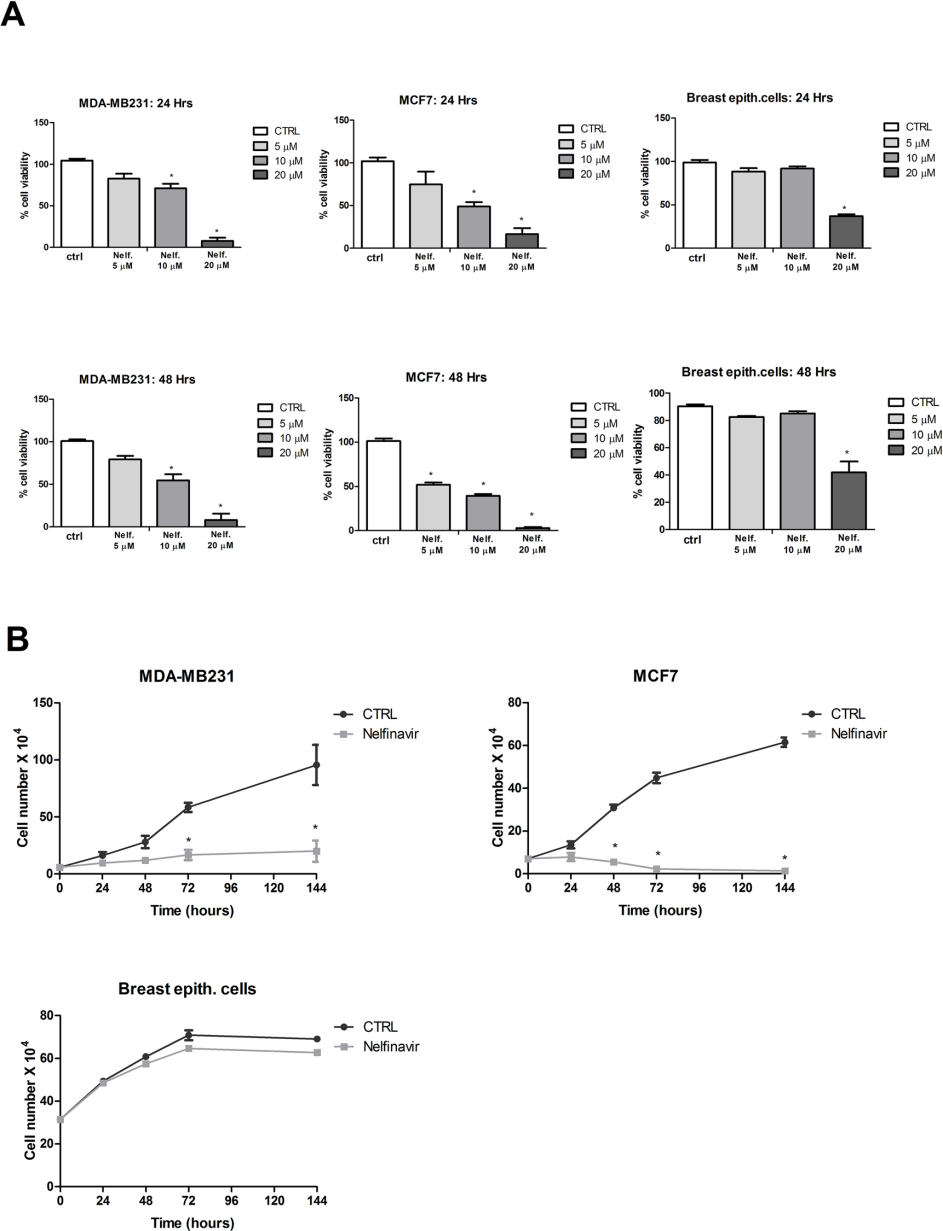
Nelfinavir exhibits anti-proliferative effects in breast cancer cell lines. **(a)** A Cell viability assay was performed in MDA-MB231, MCF-7 and normal breast epithelial cells, treated with indicated concentration of nelfinavir for 24 and 48 hours (* p < 0.05 vs ctrl). **(b)** Growth curves for 10 μM nelfinavir at indicated times in MDA-MB231, MCF-7 and breast epithelial cells. The data show the mean ± S.D. of three independent biological experiments. Significant differences in cell viability percentage were observed in cell treated with the drug compared to control cells (* p < 0.05 vs ctrl).

To determine cell viability in response to nelfinavir in human breast cancer, we performed an *in vitro* assay in MDA-MB231 and MCF-7 cells. The two tumor cell lines were treated for 24 or 48 hours with 5 μM,10 μM or 20μM of nelfinavir, doses that represent a therapeutic range in highly active antiretroviral therapy (HAART) protocols. As shown in Fig 1A, nelfinavir significantly decreased cell viability in a dose-dependent manner in both cell lines, although MCF-7 resulted more sensitive to nelfinavir treatment compared with the triple negative MDA-MB231 cell line. In addition, the drug affected the viability of primary breast normal cells only at high concentration (20 μM). The analysis of MDA-MB231, MCF-7 and breast primary normal cells proliferation confirmed nelfinavir (10 μM) selectivity in terms of anti-proliferative activity against tumor cells (Fig 1B). For this reason all the following experiments were performed with 10 μM nelfinavir.

### Cell-cycle distribution and cell-death analysis in nelfinavir-treated breast cancer cells

**Fig 2:**
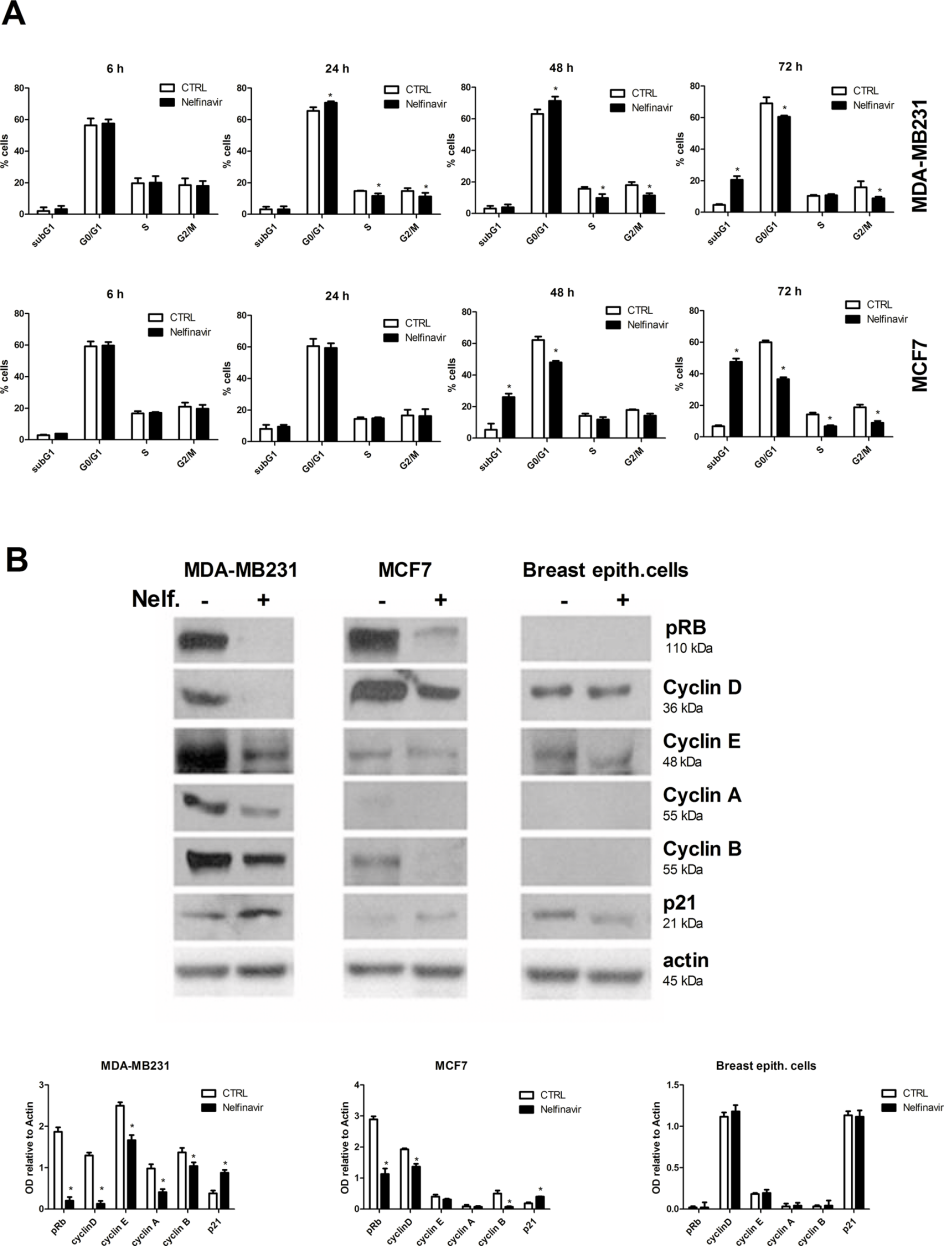
Cell-cycle analysis in nelfinavir treated breast cancer cell lines. **(a)** MDA-MB231 and MCF-7 cells were treated with nelfinavir (10 μM) for 6–72 hours. Thereafter, the cells were washed, fixed and stained with PI, and analyzed for DNA content by flow cytometry. These data represent the mean ± S.D. of four independent biological experiments (* p< 0.05 vs control). **(b)** MDA-MB231, MCF-7 and primary breast epithelial cells were treated with 10 μM nelfinavir for 24 hours and protein lysates immunoblotted for different cell-cycle regulators: pospho-Rb, cyclin A, B, D, E, p21 and beta-actin, used as loading control. Densitometric analysis of proteins signals relative to actin signal was represented. The values represent the means ± S.D. of three independent biological experiments and compared to control value (* p < 0.05 vs ctrl).

**Fig 3:**
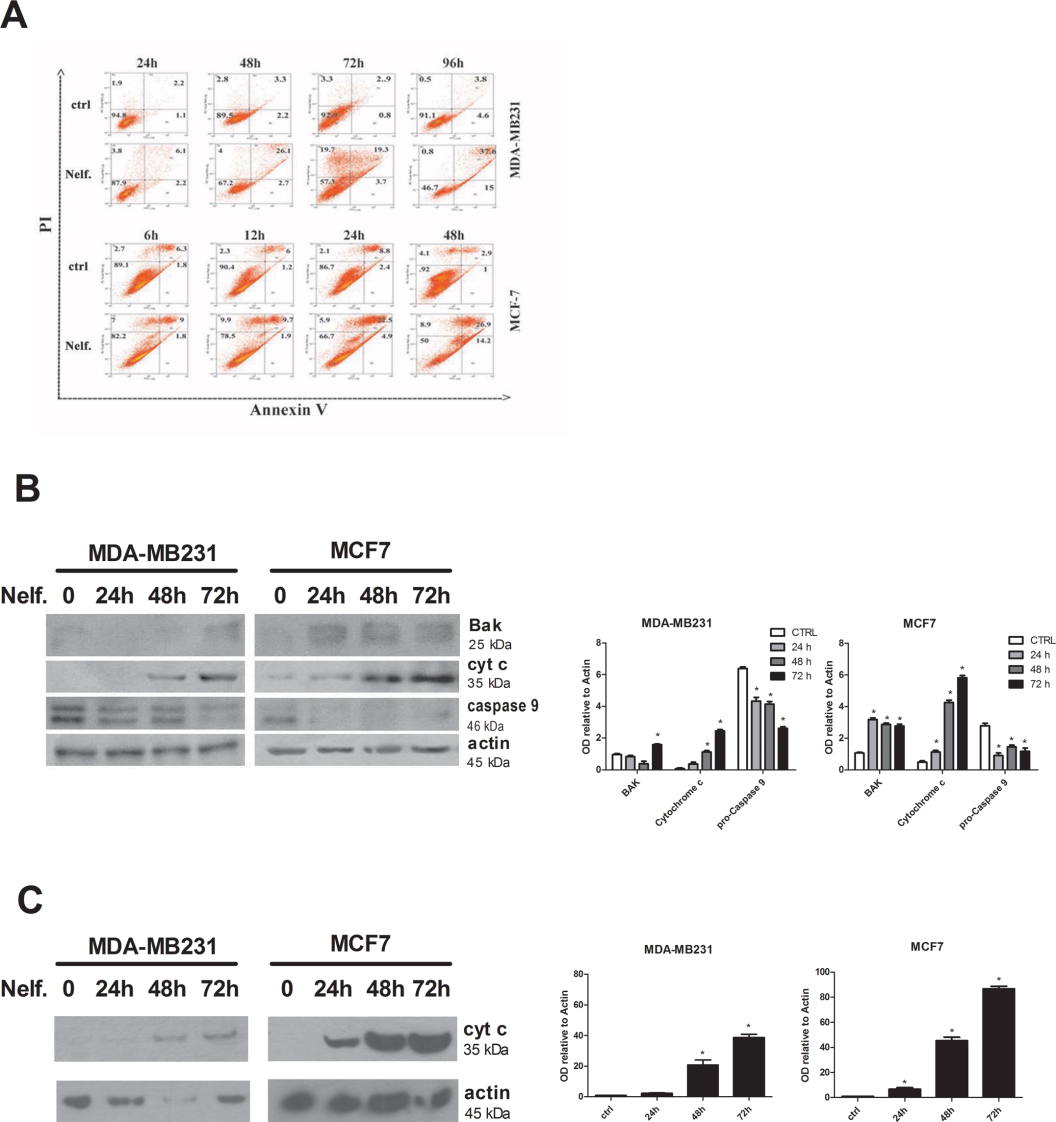
Nelfinavir induces necrosis and apoptosis in breast cancer cell lines. **(a)** MDA-MB231 and MCF-7 cells were treated with 10 μM nelfinavir for indicated time points and cells were subsequently stained with FITC-conjugated annexin V and PI and analyzed by flow cytometry. **(b)** Western blot analysis was performed in MDA-MB231 and MCF-7 treated with 10 μM nelfinavir at indicated time points and bak, cytochrome c and pro-caspase 9 proteins were revealed using specific antibodies. Beta-actin immunoblotting was used as loading control. Densitometric analysis of proteins signals relative to beta-actin signal was shown. The present data represent the means ± S.D. of three independent biological experiments and compared to control value (* p< 0.05 vs ctrl). **(c)** The release of cytochrome c in cytosolic extracts after mitochondria removal was evaluated by western blot in MDA-MB231 and MCF-7 treated with 10 μM nelfinavir at indicated time points and. Beta-actin immunoblotting was used as loading control. Densitometric analysis of proteins signals relative to beta-actin signal was shown. The present data represent the means ± S.D. of three independent biological experiments and compared to control value (* p< 0.05 vs ctrl).

The inhibition of cell-growth/viability in nelfinavir treated tumor cells could be the result of different biological mechanisms such as cell cycle arrest, apoptosis, necrosis and senescence. First, we evaluated cell cycle distribution in MDA-MB231 and MCF-7 cells. FACS analysis revealed that nelfinavir induced a slight increase of G1 phase population percentage and decrease of S and G2 phase cell percentage after 24 hours of treatment in MDA-MB231 cells. By contrast, cell-cycle arrest in G1 phase did not occur in nelfinavir treated MCF-7 cells (Fig 2A). To better investigate the drug-mediated induction of cell-cycle arrest, we performed a Western blot analysis of different proteins involved in cell-cycle progression control, such as retinoblastoma (Rb), p21, cyclins A, B, D, E. As shown in Fig 2B, nelfinavir reduced the levels of cyclin A, cyclin B, cyclin D, phosphorylated Rb, and increased the expression levels of p21 in breast cancer cell lines but not in normal cells. According with the literature, hyperphosphorylated Rb is characteristic of proliferating cells in the S and G2/M phases and the basal levels of cyclin A and E are usually undetectable in normal breast cells [61, 62]. We also demonstrated that the expression of cyclin E was modified by nelfinavir only in MDA-MB231 cells. Although both cancer cell lines have similar protein expression profiles, the biological effects of nelfinavir treatment resulted different in MDA-MB231 and MCF-7 cells. Indeed, whereas in MDA-MB231 we observed a Go/G1 block, MCF-7 cell-cycle was not affected by nelfinavir. Cell-cycle analysis also revealed that nelfinavir increased the fraction of tumor cells with sub-G1 DNA content. Therefore, we investigated whether this result, as well as the reduction of cell growth/viability, was correlated to cell death mechanisms. To this aim, MDA-MB231 and MCF7 were treated with 10 μM nelfinavir or left untreated for different time points as indicated in the Fig 3A, and stained with Annexin V conjugated with FITC and PI.

The lower left quadrant shows the viable cells (Annexin V-/PI-); the lower right quadrant represents the early apototic cells (Annexin V+/PI-); the upper right quadrant represents nonviable, late apoptotic/necrotic cells (Annexin V+/PI+); the upper left quadrant shows nonviable necrotic cells/nuclear fragments (Annexin V-/PI+).

Nelfinavir treatment induced a time-dependent increase in the proportions of apoptotic and necrotic cells (Fig 3A). In particular, nelfinavir rapidly induced necrosis followed by apoptotic process in both cell lines. A comparison between death profiles of these cell lines pointed out different cell death timetables. Indeed, 12 hours of nelfinavir treatment increased to 20% the cell-death percentage, achieving about 50% of necrotic and apoptotic cells after 48 hours of treatment in MCF-7 cells. In MDA-MB231 cells, no change in the cell-death pathways occured earlier than 48 hours of nelfinavir treatment, and more than 72 hours of drug exposure were required to determine a massive increase of PI and Annexin V positive cells (50% of cell death).

To confirm the induction of cell death by nelfinavir, we tested the effects of this drug on proteins involved in cell death pathways by western blot analysis. As shown in Fig 3B, nelfinavir treated cells displayed increased expression levels of the pro-apoptotic mitochondrial factor Bak and decrease of the precursor form of caspase 9 in a time-dependent manner. Moreover, nelfinavir also induced the increase of total levels of cytochrome c (Fig 3B) that is associated with an increase of the protein in cytosolic extracts after mitochondria removal (Fig 3C). Therefore, parallel to the cytotoxic effect revealed by cell viability assay and FACS analysis, treatment of MDA-MB231 and MCF-7 cells with nelfinavir for 72 and 24 hours respectively, increased the levels of apoptotic markers. These data suggest that short-term nelfinavir treatment induced cell-death directly in MCF-7cells, and did not have a prominent cytotoxic effect in MDA-MB231, which were arrested in G0/G1 phase. However, prolonged cell-cycle arrest induced necrosis and activation of the apoptotic process. Therefore, MDA-MB231 cells seem less sensitive to nelfinavir induced cytotoxicity.

### Nelfinavir treatment downregulates Akt signaling in breast cancer cells

**Fig 4:**
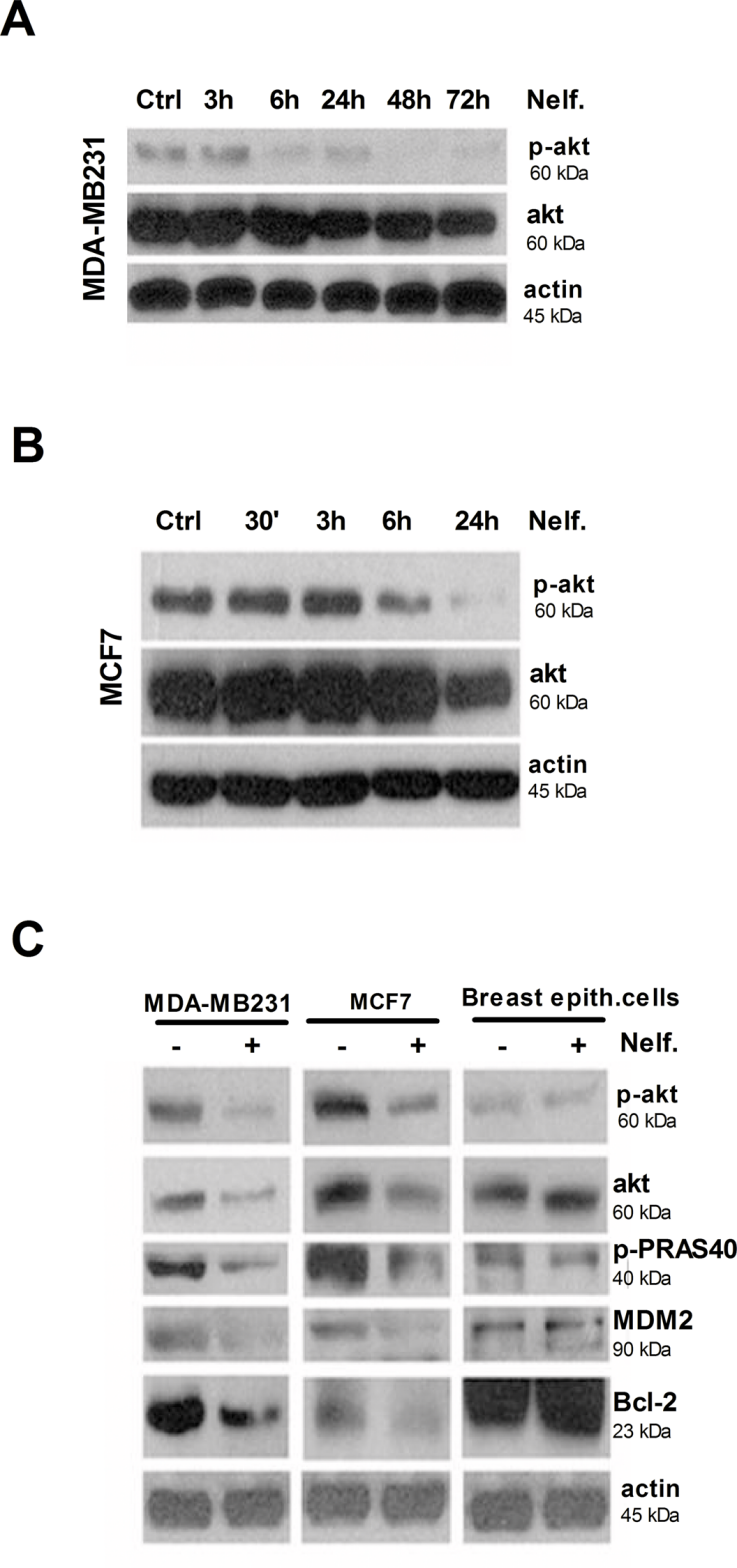
Nelfinavir inhibits Akt signaling in cancer but not in normal breast cells. Protein lysates from MDA-MB231 cells **(a)** or MCF-7 cells **(b)**, subjected to 10 μM nelfinavir treatment for the indicated time points, were immunoblotted for phosphorylated and total Akt antibodies. **(c)** MDA-MB231, MCF-7 and normal breast epithelial cells were treated with 10 μM nelfinavir and lysed after 24 hours. Protein lysates were subjected to western blot analysis of Akt and its effectors phospho-PRAS, MDM2 and Bcl-2, using specific antibodies. Beta-actin immunoblotting was used as loading control. Data are representative of three independent biological experiments.

Akt signaling pathway has been implicated in the regulation of cell cycle progression and cell proliferation. Activation of Akt is also associated with protection of cells from apoptosis [63–65]. To analyze whether the inhibition of Akt was related to nelfinavir induced cell cycle arrest and/or apoptosis, we evaluated Akt expression and phosphorylation by western blot. MDA-MB231 and MCF-7 cells were treated with nelfinavir at different time points, depending on cell-death profile: 3, 6, 24, 48, 72 hours for MDA-MB231 cells and 0,5, 3, 6 and 24 hours for MCF-7 cells. As shown in Fig 4A and 4B, treatment with nelfinavir for 6 hours determined a significant reduction of Akt phosphorylation in both cell lines. Surprisingly, also total Akt protein was downregulated following 24 hours of drug exposure, suggesting that enhanced Akt de-phosphorylation at this time point could be explained by reduction in total Akt protein levels.

To determine whether the downregulation of Akt affected downstream targets and its specificity for tumor cell lines, we analyzed the expression levels of the most representative proteins involved in Akt signaling in breast cancer and normal breast epithelial cells. Western blot analysis revealed a reduction of all analyzed Akt targets, such as phospho-PRAS, MDM2 and Bcl2, in MDA-MB231 and MCF-7 cells, while no effects were observed in normal cells (Fig 4C).

### Nelfinavir induces Akt downregulation by disruption of the Akt-HSP90 complex

**Fig 5:**
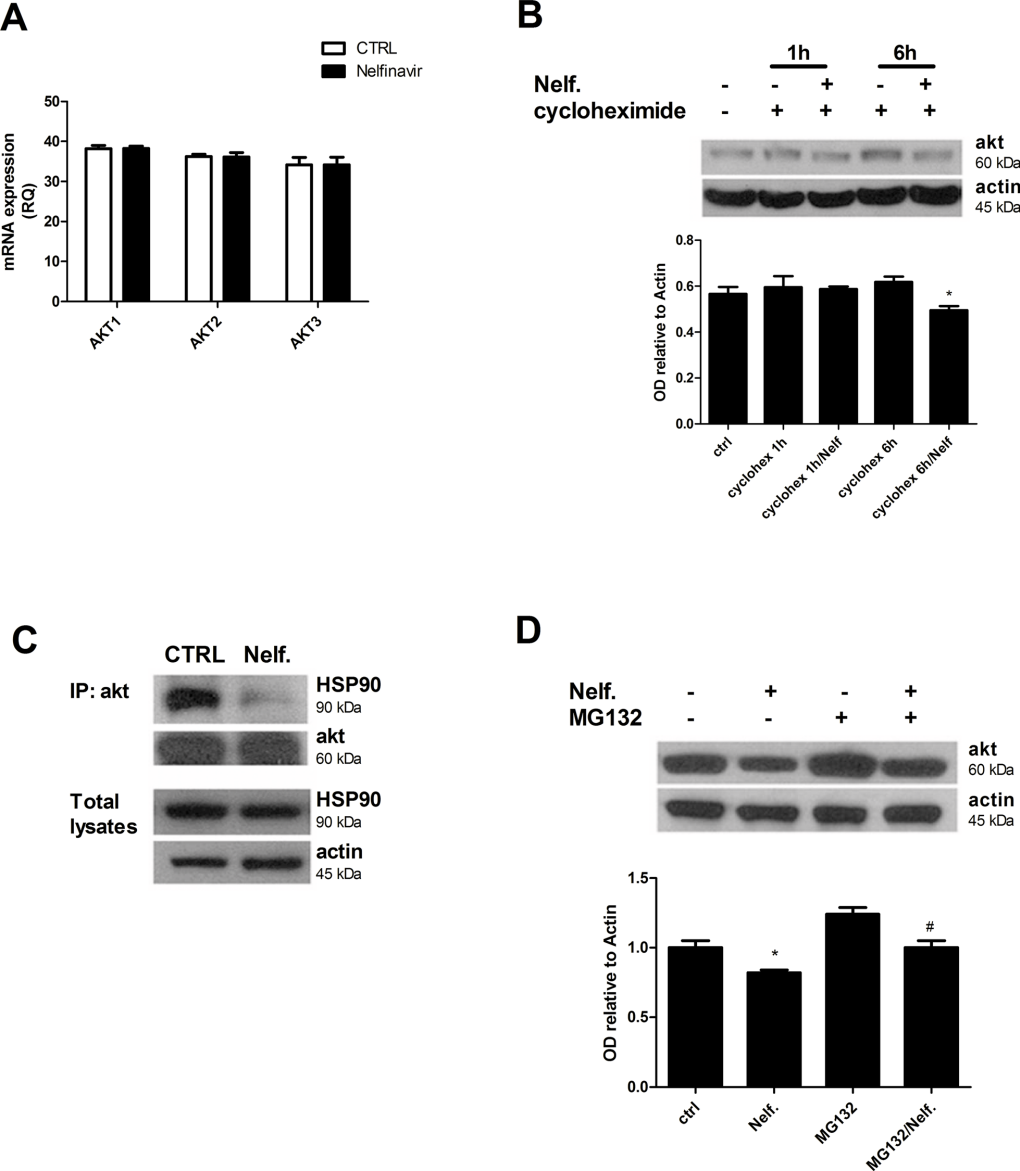
Nelfinavir causes a dissociation of Akt/HSP90 complex and Akt degradation. **(a)** MDA-MB231 cells were treated with 10 μM nelfinavir for 24 hours and mRNA expression levels of three Akt isoforms were analyzed by qRT-PCR as indicated in Material and Methods. The values represent means ± S.D. of three independent biological experiments. **(b)** MDA-MB231 cells were treated with 10 μM nelfinavir for 24 h and incubated with 0,5 μg/mL cycloheximide for the last 1 hour or 6 hours of treatment. Protein lysates were subjected to western blot analysis for Akt and beta-actin. (* p < 0.05 vs cycloheximide 6h) **(c)** Cells were treated with nelfinavir for 6 hours, then lysed, immunoprecipitated (IP) using Akt antibody and immunoblotted for HSP90 or Akt. **(d)** Lysates from MDA-MB231 cells co-treated with nelfinavir for 24 hours and proteasomal inhibitor MG132 (10 μM) for the last 8 hours of drug treatment were subjected to western blot for Akt and beta-actin. Akt signals following the indicated treatments were quantified by densitometry and normalized on beta-actin values. The values are representative of three independent biological experiments (* p < 0.05 vs ctrl; # p< 0.05 vs Nelfinavir).

In order to understand whether Akt decrease occurred at the transcriptional level, we analyzed the expression of Akt mRNA in MDA-MB231 cells by a reverse transcription-PCR experiment. As shown in Fig 5A, treatment with nelfinavir for 24 hours did not inhibit the transcription of all three Akt isoforms (Akt 1, 2, 3) mRNA. To investigate whether nelfinavir-mediated Akt regulation took place at post-transcriptional level, we analyzed Akt stability, treating MDA-MB231 cells with cycloheximide to block new protein synthesis. Fig 5B showed that nelfinavir modified Akt turnover by reducing Akt expression levels in presence of cycloheximide, when compared to single cycloheximide treatment. This result suggests that nelfinavir does not affect *de novo* protein synthesis.

Because Akt stability is mainly dependent from its association with chaperone HSP90, we evaluated the association between Akt and HSP90 by co-immunoprecipitation assay. As shown in Fig 5C, 6 hours of nelfinavir treatment reduced Akt/HSP90 association without affecting Akt and HSP90 expression levels. Nelfinavir mediated disruption of HSP90/Akt complex could explain the significant and fast de-phosphorylation of Akt, as well as the downregulation of total Akt. To determine whether nelfinavir induces Akt degradation, and whether proteasome mediates this process, cells were treated with the proteasome inhibitor MG132, and Akt was detected by western blot analysis (Fig 5D). The proteasome inhibitor impaired nelfinavir effects restoring Akt protein levels, thus suggesting that nelfinavir could induce Akt degradation at least in part via proteasome involvement.

### Nelfinavir induces the increase of ROS production and lipid peroxidation in breast cancer but not in normal cells

**Fig 6:**
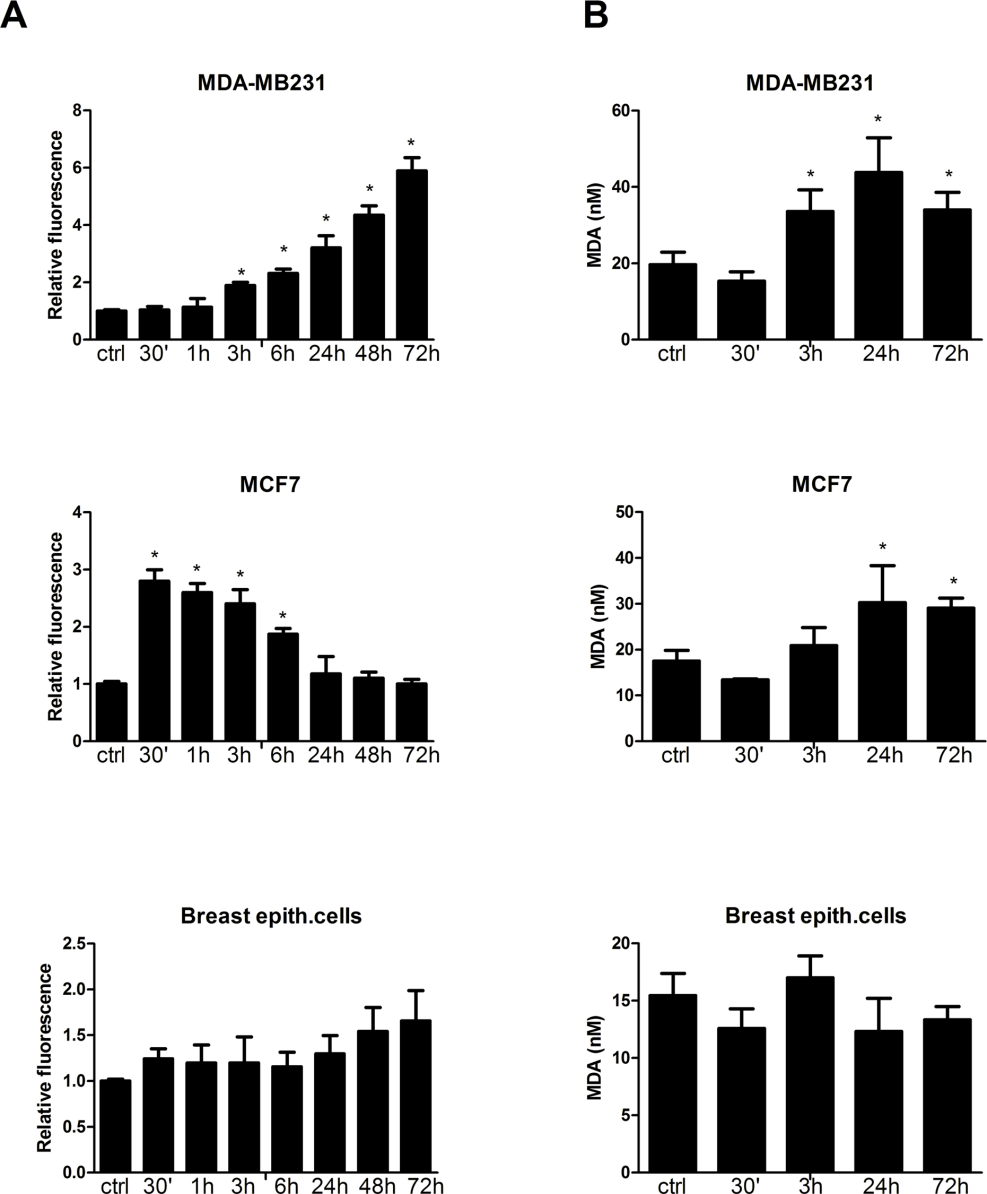
Nelfinavir induces ROS accumulation and lipid peroxidation in breast cancer cells. **(a)** MDA-MB231, MCF-7 and primary breast epithelial cells were subjected to 10 μM nelfinavir treatment for 30 minutes-72 hours. ROS production was measured by H2DCF-DA staining and fluorescence intensity was expressed as MFI normalized to untreated cell values. Each value is the mean ±S.D. of three different biological experiments (*p< 0.05 vs ctrl). **(b)** Cells were treated with 10 μM nelfinavir for the indicated time points, and processed as indicated in Material and Methods. Colorimetric analysis revealed the concentration of MDA (nM), a lipid peroxidation marker. The present data derived from three different biological experiments (*p< 0.05 vs ctrl).

The degradation of Akt protein and the presence of high percentage of necrotic cells in nelfinavir-treated cells suggested an involvement of a fast-acting mechanism such as reactive oxygen species. To assess ROS production in these cells, we performed a FACS analysis through the observation of H2DCF-DA oxidation. Nelfinavir induced time-dependent production of ROS with a different trend in the analyzed cell-lines (Fig 6A). The increase of ROS production was rapid in MCF-7 cells, starting at 30 minutes, and progressively reduced until 24 hours of nelfinavir treatment. MDA-MB231 cells treated with this drug exhibited a slight increase of intracellular ROS levels within 3 hours, with a gradually enhancement in a time-dependent manner. These different trends of ROS level changes reflected also the cell-death induction observed in these two cell lines. Therefore, high levels of ROS at 30 minutes in MCF7 cells could explain the earlier cell-death induction in MCF-7 cells compared to MDA-MB231 cells. In both cell lines, a massive ROS production rapidly caused necrosis, while a slight increase of ROS levels occurring in the second part of the time course was able to regulate apoptotic pathways. By contrast, in normal primary breast cells, only long term nelfinavir treatment induced a not statistically significant increase of ROS production.

Since ROS cause macromolecular damage by a rapid attack to the polyunsatured fatty acids of the membrane, we investigated whether nelfinavir induced lipid peroxidation. To this aim, MCF-7 and MDA-MB231 cells were treated with 10 μM nelfinavir at different time points and the concentration of lipid peroxidation malondialdehyde (MDA) was quantified by colorimetric assay. As depicted in Fig 6B, while in MDA-MB231cells nelfinavir induced a progressive increase of lipid peroxidation starting from 3 hours up to 72 hours, in MCF-7 cells this effect began at 24 hours of treatment. No significant modification of lipid oxidation status was observed in normal cells. This assay highlights an early detoxifying response to the perturbation of the redox state induced by the drug, suppressed after 3 hours in tumor cells. These data suggest a protective response of normal cells to nelfinavir-induced oxidative stress, whereas breast cancer cell lines showed an impaired detoxifying capability.

### ROS mediate the disruption of the Akt-HSP90 complex and the induction of cell-death in nelfinavir-treated cancer cells

**Fig 7:**
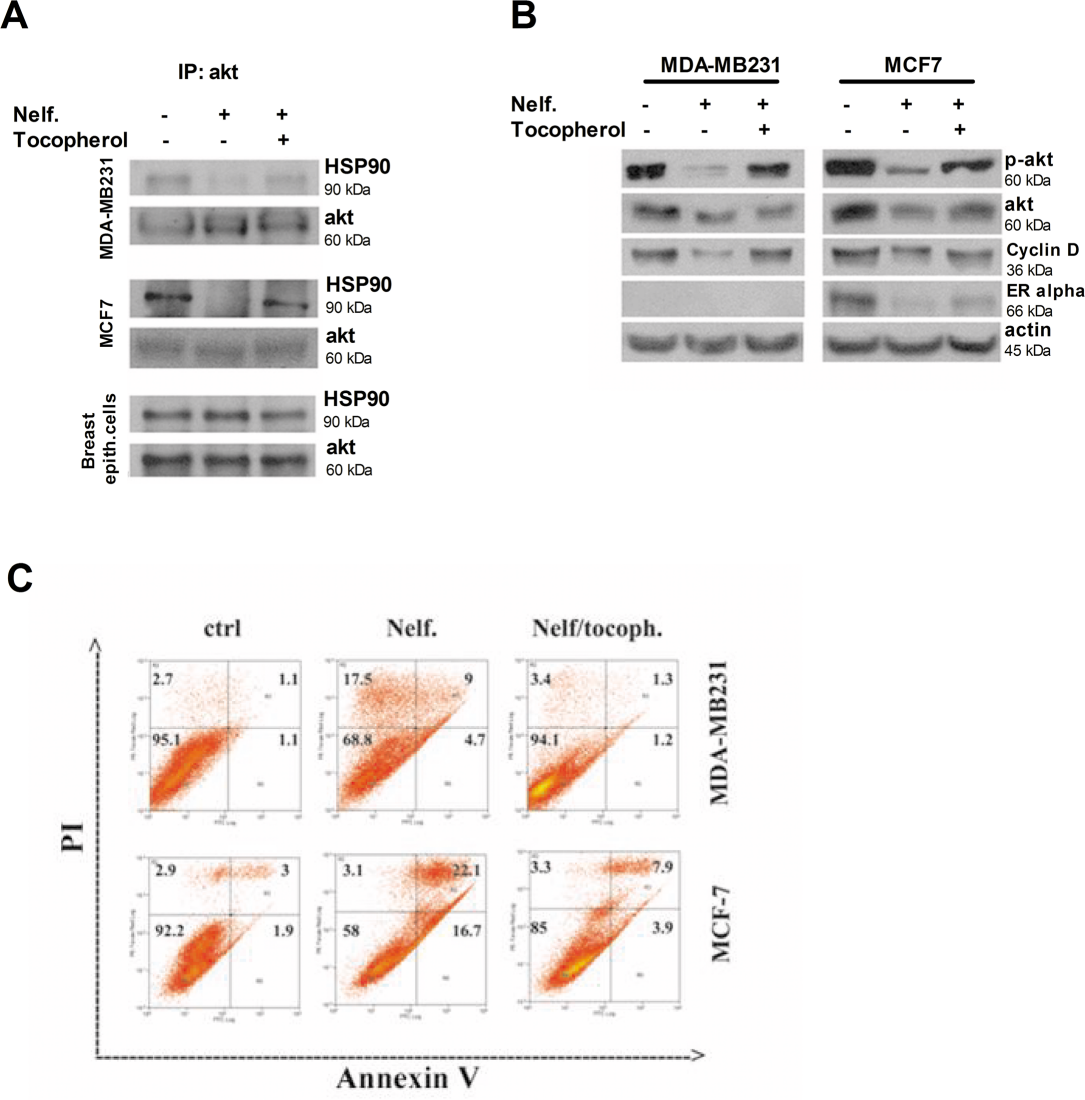
The nelfinavir-induced Akt/HSP90 complex disruption and tumor cell-death are ROS-mediated. **(a)** MDA-MB231, MCF7 and breast epithelial cells were treated with 10 μM nelfinavir and 35 μM tocopherol for 6 hours, and equal amounts of protein lysate were immunoprecipitated using Akt antibody followed by immunoblotting with anti-HSP90 and anti-Akt. **(b)** MDA-MB231 and MCF-7 cells were treated with 10 μM nelfinavir and 35 μM tocopherol for 24 hours and phospho-Akt, Akt, cyclin D, ERα, beta-actin levels were monitored using the respective antibody by western blot on total lysate. Beta-actin immunoblotting was used as a loading control. **(c)** MDA-MB231 and MCF-7 cells were treated with nelfinavir (for 72 and 24 hours respectively) in the absence or presence of 35 μM tocopherol. Cell-death profile was examined by cytofluorimetric analysis of annexin V/ PI positivity. The cell percentage were reported in corresponding areas of dot-plot. Three different biological experiments confirmed these cell distributions.

In physiological conditions, ROS are very important regulators of many intracellular pathways such as cell proliferation and metabolism, but at higher concentration they can determine the opposite effects by blocking survival pathways and inducing apoptosis and necrosis. To investigate whether ROS were responsible for Akt downregulation and Akt/HSP90 complex dissociation, breast cancer cells were treated with nelfinavir in the presence of the antioxidant tocopherol, and the HSP90/Akt complex was co-immunoprecipitated for western blot analysis. As shown in Fig 7A, in breast cancer cell lines, the presence of tocopherol impaired nelfinavir-induced disruption of Akt/HSP90 complex. This result suggests that ROS production is an earlier event than Akt downregulation. According to the literature, this result indicates a relevant correlation between high intracellular ROS levels and Akt/HSP90 downregulation [66, 67]. Because ROS promoted the disruption of the Akt/HSP90 complex in tumor cells treated with nelfinavir, we also evaluated the protein expression levels of two other HSP90 clients such as cyclin D and ERα. Fig 7B shows that these proteins were reduced by nelfinavir treatment, whereas the addition of tocopherol restored their expression levels at those of untreated cells. To confirm the primary role of ROS in the mechanism of action of nelfinavir, we assessed the capability of tocopherol to prevent nelfinavir induced cell death. As expected, the presence of tocopherol in tumor cells treated with nelfinavir impaired ROS overproduction and subsequent apoptotic and necrotic processes (Fig 7C).

### Nelfinavir perturbs cell redox state by affecting ROS-scavengers and ROS-producer enzymes

**Fig 8:**
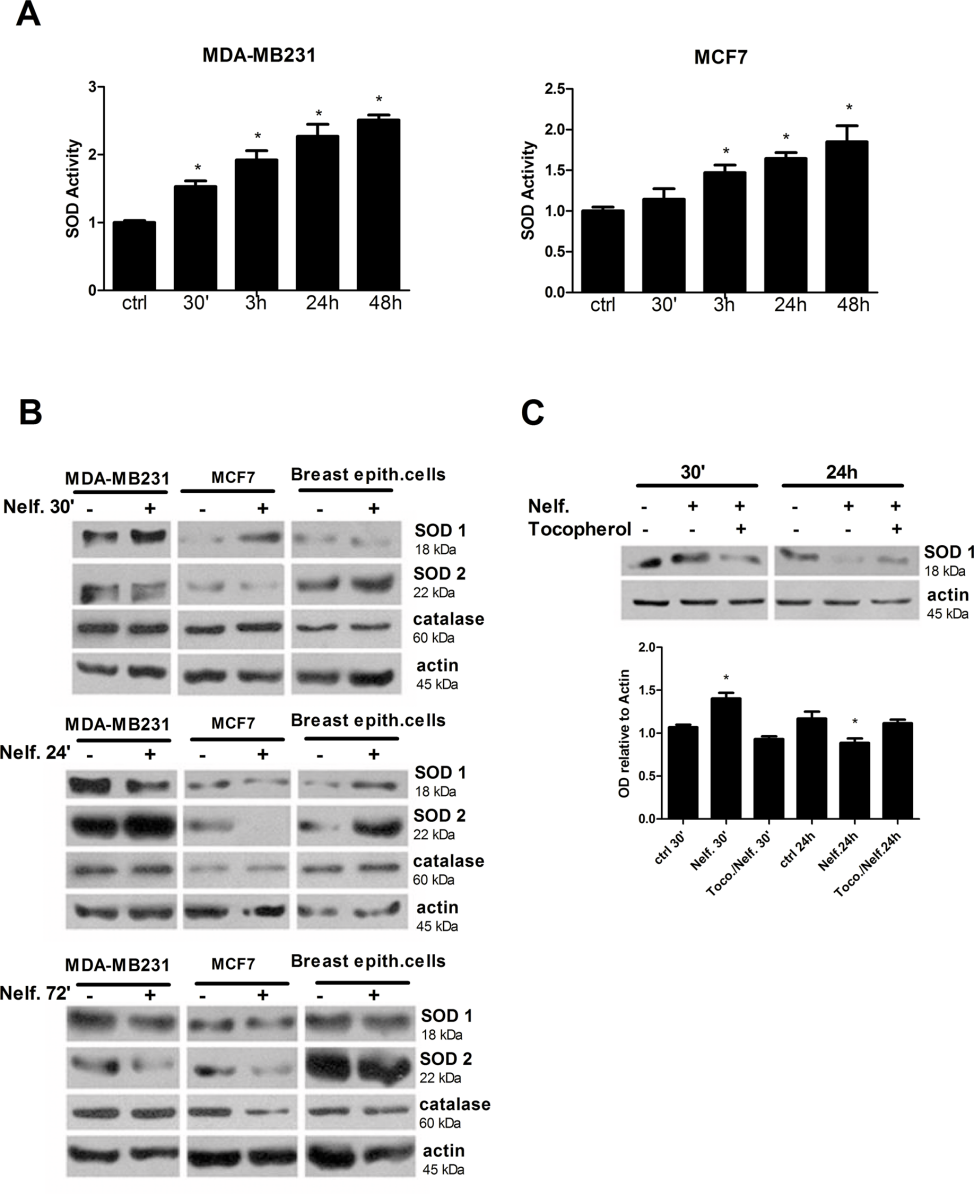
Nelfinavir regulates SOD activity and expression in a time-dependent manner. **(a)** Breast cancer cells were treated with 10 μM nelfinavir at indicated times and SOD activity (relative to activity of untreated cells) was analyzed as indicated in Material and Methods. Values are representative of three independent biological experiments ± S.D., (*p< 0.05 vs ctrl). **(b)** Protein lysates derived from MDA-MB231, MCF-7, or breast epithelial cells, treated with 10 μM nelfinavir for the indicated time points, were immunoblotted with anti- SOD1, anti-SOD2 and anti-catalase. Beta-actin was used as loading control. **(c)** MCF-7 cells, treated with 10 μM nelfinavir ± 35 μM tocopherol for 30 minutes or 24 hours, were lysed and subjected to western blot analysis for SOD1. Beta-actin was used as loading control. Densitometric analysis of SOD1 signal relative to beta-actin signal was represented. The values represent the means ± S.D. of three independent biological experiments and are compared to control value (*p< 0.05 vs ctrl).

To better investigate redox state alterations induced by nelfinavir in breast cancer cells, and to identify the source of ROS production, we analyzed the activity of the main ROS detoxifying-enzyme, SOD. In MDA-MB231 and MCF-7 cells, nelfinavir increased SOD activity in a time dependent manner compared with control (Fig 8A). Since SOD acts both as antioxidant enzyme for removing superoxide anion and as ROS inducer for production of hydrogen peroxide, the increase of SOD activity could be a pro- and anti-oxidant condition. For this reason, the enhanced SOD activity after the treatment with nelfinavir could represent a source of ROS production as well as the effect of oxidative stress response. To further investigate the role of SOD and the involvement of catalase in oxidative stress induction, their expression levels were analyzed following nelfinavir treatment in breast cancer and normal breast epithelial cells. Western blot analysis revealed a time and cell-type dependent regulation of SOD1 and SOD2 expression levels by nelfinavir, while catalase levels did not change following nelfinavir treatment (Fig 8B). Importantly, cells more sensitive to oxidative stress such as MCF-7 showed a lower basal levels of catalase compared to MDA-MB231 and normal breast cells, suggesting a protective role of catalase against drug-induced oxidative damage. At an early time of 30 minutes of nelfinavir treatment, SOD1 was upregulated in MDA-MB231 and in MCF7 cells, while SOD2 levels increased following 24 hours of treatment. Although also normal cells showed an increase of SOD1 and SOD2 expression at an early time of nelfinavir treatment, long term treatment did not affect the levels of SOD1 and SOD2. On the other hand, in tumor cell lines the early increase of SOD1 and SOD2 levels was followed by a strong reduction of both protein expression after 72 hours of treatment (Fig 8B). These data suggest an involvement of SOD1 at an early step of nelfinavir anticancer activity.

To determine whether SOD1 upregulation at this early stage was responsible for the increase of ROS production or rather it represented a ROS-induced effect, breast cancer cells were treated with tocopherol, and SOD expression analyzed by western blot. As shown in Fig 8C, both the upregulation of SOD1 after 30 minutes of drug treatment and its reduction at 24 hours were dependent by ROS production, since the antioxidant tocopherol restored basal SOD1 expression levels. These data suggested that SOD1 and SOD2 did not act as ROS-producers but rather their activity and expression levels were regulated by reactive species following the treatment with nelfinavir.

Although the majority of cellular ROS production originates from mitochondria, flavoenzymes represent the key modulators to generate highly regulated amounts of ROS including superoxide, hydrogen peroxide and nitric oxide [68].

**Fig 9:**
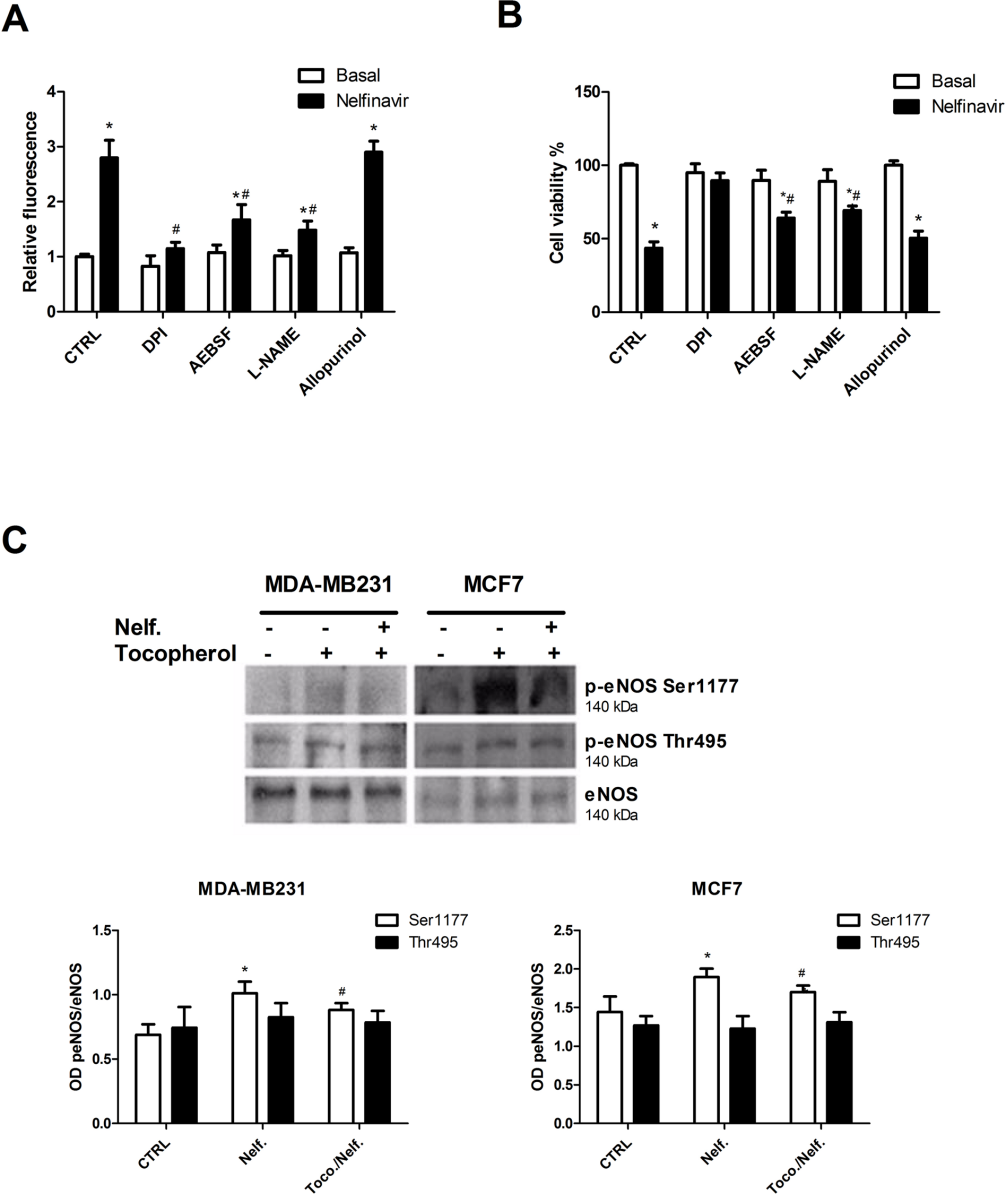
Nelfinavir-mediated ROS increase and cytotoxicity depend on flavoenzyme activation. **(a)** MCF-7 cells were treated with or without Nelfinavir (10 μM) for 30 minutes after pre-incubation with DPI (20 μM), AEBSF (40 μM), L-NAME (5 mM) or allopurinol (100 μM). The different cell conditions were analyzed for ROS content by FACS measurement of H2DCF-DA. Each value represent mean of three biological experiments ± S.D. normalized on nelfinavir-untreated cells value (*p< 0.05 vs basal; # p< 0.05 vs Nelfinavir). **(b)** A similar experiment was performed using nelfinavir for 24 hours in MCF-7 cells pre-treated with DPI (20 μM), AEBSF (40 μM), L-NAME (5 mM) or allopurinol (100 μM), and MTT-derived staining intensities were analyzed by a photometer. Bar graphs report the values of mean of three independent biological experiments ± S.D., normalized on nelfinavir-untreated cells value (*p< 0.05 vs basal; # p< 0.05 vs Nelfinavir). **(c)** MDA-MB231 and MCF-7 cells were treated with nelfinavir for 30 minutes in the absence or presence of 35 μM tocopherol, then lysed and subjected to western blot for eNOS, phopho-eNOS(Ser1177) and phospho-eNOS (Thr495). Densitometric analysis of proteins signals relative to eNOS signal was shown. The values display the means ± S.D. of three independent biological experiments and are compared to control value (*p< 0.05 vs ctrl; # p< 0.05 vs Nelfinavir).

To determine the source of ROS involved in nelfinavir oxidative effect, we analyzed the contribution of the main cytosolic flavoenzymes such as NADPH Oxidase (NOX), Xantine Oxidase (XO) and eNOS. As shown in Fig 9A, the pre-treatment with DPI, a non-specific flavoenzymes inhibitor [69], impaired the nelfinavir related ROS enhancement. Moreover, the use of specific inhibitors revealed that the pre-treatment with NOX or eNOS inhibitors, AEBSF and L-NAME respectively, prevented the nelfinavir mediated full increase of ROS, suggesting an important involvement of these two ROS-producers in nelfinavir mechanism of action. On the contrary, the suppression of XO activity with allopurinol did not affect nelfinavir modulation of oxidative status. The important role of NOX and eNOS in nelfinavir mediated effects were supported by cell viability assay where MCF7 cells were pre-treated with flavoenzymes inhibitors in presence of nelfinavir. We demonstrated that inhibition of NOX or eNOS activity prevented nelfinavir mediated citotoxicity in breast cancer cells (Fig 9B). Given such results, we then investigated the effect of nelfinavir treatment in the activation of eNOS by western blot analysis of total and phosphorylated forms of eNOS. Results of Fig 9C demonstrate for the first time that nelfinavir induced eNOS activation via phosphorylation of Ser1177 in breast cancer cells, and the addition of tocopherol reduced at least in part nelfinavir-mediated e-NOS phosphorylation. In the same condition, the levels of eNOS and phospho-eNOS(Thr495) were not modified (Fig 9C).

## Discussion

Actually, Nelfinavir is known to have anti-cancer activity in different tumor types including breast cancer [8, 23, 70]. Among breast cancer cell lines, nelfinavir is effective in different cell lines independently from their genetic profile (HER2, ER, PR, TP53). Here we evaluated the molecular signaling that is activated by nelfinavir treatment in two HER2 negative breast cancer cell lines (MCF-7 and MDA-MB231), based on previous works which have already demonstrated its effectiveness in these kind of cell [11, 71, 72]. Here, we validated the anti-proliferative and specific cytotoxicity of nelfinavir in these cell lines, demonstrating that 10 μM of nelfinavir, was able to reduce tumor cell viability without affecting normal primary breast cell-viability. At the same concentration, nelfinavir induced cell-cycle arrest in G0/G1 phase, and subsequently determined cell death in MDA-MB231, whereas it mainly caused direct cell death in MCF-7 cells. As suggested by previous works [61], the different basal levels of expression of cyclins A and E, and the significant nelfinavir-induced reduction of cyclin E limited to MDA-MB231 cells, could play a role in the different responses of cell lines to nelfinavir in cell-cycle progression. In both cell lines, nelfinavir caused a fast induction of necrosis, suggesting a direct cytotoxic effect. Triple-negative breast cancer (MDA-MB231) tends to be more aggressive and is correlated with worse prognosis than receptor-positive subtypes (MCF-7). Their genetic profile confers them a high resistance to treatments, as we also observed in response to nelfinavir. Indeed, besides the effectiveness of nelfinavir in MDA-MB231, these cells are less sensitive to nelfinavir induced cytotoxicity compared with MCF-7 cells.

It has been well established that inhibition of Akt phosphorylation is an important mechanism by which nelfinavir exerts antitumor activity in several cancer types [8, 12, 18], although its involvement in breast cancer has not been elucidated. Our results demonstrated for the first time that nelfinavir inhibits AKT phosphorylation through the regulation of total AKT levels. Such regulation did not occurr at transcriptional level, but rather nelfinavir enhanced Akt protein turnover by reducing Akt/HSP90 association. Indeed, when dissociated from its chaperon, Akt became more sensitive to protein phosphatase 2A-mediated dephosphorylation and consequently to the degradation by the proteasome [73]. In line with these observations, the use of MG132 confirmed that nelfinavir induces Akt degradation, since this proteasome inhibitor impaired nelfinavir effects on Akt. However, we cannot exclude the involvement of other mechanisms of protein degradation. Contrary to the hypothesis of HSP90 as primary nelfinavir target [25], we demonstrated that Akt/HSP90 disruption is dependent upon nelfinavir-induced oxidative stress. Several evidences allow to hypothesize that ROS could play a primary role in anticancer activity of nelfinavir: a) the presence of high necrotic cell percentage following nelfinavir short-term treatment in breast cancer cells; b) the observation that HSP90 is sensitive to oxidation [66, 67]; c) clinical studies on nelfinavir as anti-viral drug that revealed an involvement of ROS in side effects onset [74–78]. We demonstrated that a short exposure to nelfinavir led to a massive ROS production causing necrosis, while a slight increase of ROS levels occurring after a prolonged exposure seemed to regulate apoptotic pathways, as also suggested by previous studies [79–81]. The observation of ROS-generating capability of nelfinavir was further supported by the increase of lipid peroxidation. To investigate whether ROS are responsible of Akt downregulation and Akt/HSP90 complex dissociation, cells were treated with the antioxidant tocopherol. Tocopherol impaired nelfinavir-induced disruption of the Akt/HSP90 complex in breast cancer cells, and had no effects in normal cells, suggesting that ROS production is essential for the regulation of AKT signaling in breast cancer cells. Previous reports show that HSP90 can be cleaved by ROS in a highly conserved N-terminal aminoacid motif, resulting in the loss of its chaperon function and the degradation of its client proteins in cancer cell lines [66, 67]. Accordingly, we reported that nelfinavir was able to disrupt Akt/HSP90 complex in MCF-7 and MDA-MB231 cells but not in primary normal breast cells.

In addition, the use of antioxidant in cells treated with nelfinavir impaired apoptosis and necrosis processes, thus confirming the primary role of ROS in the mechanism of action of nelfinavir. It is plausible that oxidative stress produced by nelfinavir affects other pathways in addition to Akt/HSP90 signaling to cause cell death. Behind Akt, other HSP90 clients resulted downregulated by the treatment with nelfinavir such as ERα and cyclin D, supporting the hypothesis that many oncogenic kinases, HSP90 clients, such as Raf-1, Bcr-Abl and HER2 [82], could be regulated by the drug. It has been well established that human breast tumor cell lines produce more ROS than non-transformed cells [48–50], in order to promote genome instability and alterations in cell signaling processes [45, 47, 83, 84], but they are more sensitive than non-transformed cells to cytotoxic oxidative stress [85, 86]. The different behavior between cancer and normal cells could be related to loss of activity/ impairment of antioxidants [87–90] or to an upregulation of ROS-producer enzymes [68, 91, 92]. Here we demonstrated that nelfinavir regulated SOD activity and its expression levels by ROS production in breast cells. Long-term treatment with nelfinavir reduced protein levels of SOD1, SOD2 and catalase in tumor cells, and while it was well established that detoxifying enzymes can be activated and upregulated by ROS [43], their decrease in oxidative stress condition is not completely understood. The reduction of antioxidant enzyme could be due to free radicals-mediated direct damage [39, 93, 94] or to downregulation of Akt signaling [95]. Our analysis of the main cytosolic ROS-producer enzymes revealed that nelfinavir modulated flavoenzymes activity, since the pre-treatment with non-specific flavoenzyme inhibitor DPI [69] impaired nelfinavir-mediated ROS enhancement. Moreover, a specific inhibition of eNOS or NOX prevented the full increase of ROS. Cell viability assay performed in the same experimental condition confirmed the involvement of eNOS and NOX in nelfinavir cytotoxicity. Furthermore, nelfinavir induced eNOS activation via phosphorylation of Ser1177 [96] also in breast cancer cells, an effect at least in part impaired by tocopherol, suggesting an important but not prominent role of this enzyme in nelfinavir mechanism of action. It remains to clarify whether flavoenzymes represent nelfinavir primary targets. It is established that HIV protease inhibitors while substantially improve life expectancy and quality of life in HIV-positive patients, their long-term usage can initiate toxic side-effects that may lead to cardio-metabolic dysfunction, such as increased ROS production [27]. However, in a different context an increase of ROS production represents the main and necessary event. Indeed, nelfinavir exerts an anticancer effect by inducing ROS production, taking advantage of different redox balance regulation between cancer and normal cells. This suggests nelfinavir-induced cytotoxic oxidative stress as an effective therapeutic strategy for cancer encouraging the design of novel and more effective nelfinavir-derivatives for pre-clinical and clinical applications.
